# Polarization of Macrophages in Epidural Inflammation Induced by Canine Intervertebral Disc Herniation

**DOI:** 10.3389/fvets.2020.00032

**Published:** 2020-01-31

**Authors:** Núria Vizcaíno Revés, Helga Maria Mogel, Michael Stoffel, Artur Summerfield, Franck Forterre

**Affiliations:** ^1^Department of Clinical Veterinary Medicine, Vetsuisse Faculty, University of Bern, Bern, Switzerland; ^2^Division of Veterinary Anatomy, Vetsuisse Faculty, University of Bern, Bern, Switzerland; ^3^Institut für Virologie und Immunologie, Institut für Infektionskrankheiten und Pathobiologie, Universität Bern, Bern, Switzerland

**Keywords:** canine, intervertebral disc, macrophages, polarization, disc degeneration

## Abstract

**Introduction:** Canine interverterbral disc (IVD), although physiologically acellular, displays an inflammatory cell population consisting almost exclusively of macrophages (Mϕ) when acutely herniated. Mϕ encompass a heterogenous cell population, roughly divided into classically (M1) or alternatively activated (M2)Mϕ. Polarization into M1 Mϕ leads to strong antimicrobial activity and pro-inflammatory response. In contrast, M2Mϕ exibit anti-inflammatory function and regulate wound healing. The purpose of this study was to characterize the phenotype of the Mϕ population present in naturally occurring IVD herniation.

**Materials and Methods:** IVD material of dogs with IVD disease was collected during standard decompressive surgery. A negative control consisting of IVD material of dogs without IVD degeneration and a positive control consisting of canine liver and lymph node samples were also included. All samples were embedded in OCT and shock frozen. Eight micrometer cryostat sections were prepared, air dried and immunostained without prefixation or permeabilization. CD14 was used as marker Mϕ, MHCII for M1Mϕ and CD206 for M2Mϕ.

**Results:** Fifteen samples of dogs with IVD herniation, 10 negative, and 5 positive control samples were obtained. No positive cell was found in the negative control group. The positive control group displayed several MHCII and CD206 positive cells, all of them being simultaneously positive to CD14. All herniated samples displayed a mixed population of M1Mϕ and M2Mϕ, and some sparse Mϕ displaying markers for both M1 and M2Mϕ simultaneously.

**Conclusion:** The mixed phenotype encountered shows the plasticity and dynamism of Mϕ and evidences the chronic component of IVD disease despite its acute clinical presentation.

## Introduction

Intervertebral disc (IVD) herniation is one of the most common neurological diseases in dogs, and results in clinical signs that range from hyperesthesia to plegia without nociception.

While the research focus on IVD disease has historically been centered over the clinical and pathological effects of IVD extrusion on the spinal cord, the interest on the pathological changes in the epidural space has rose the last few years (1–4).

The canine nucleus pulposus, an anatomic region of the IVD which under physiologic circumstances consists of very low numbers of chondrocyte-like cells responsible for secreting the extracellular matrix has been shown to display a cell population consisting of inflammatory cells, mostly macrophages (Mϕ) when herniated (5–7). Similarly, an immunophenotype of inflammatory response consisting of CD68-positive cells likely representing differentiation from monocytes to macrophages was also reported in a study that collected human herniated IVD material (8).

Macrophages are phagocytic cells derived from monocyte precursors. They mediate the immune response contributing to both initiation and resolution of inflammation. Following tissue injury, macrophage precursors are recruited via chemokine gradients. These macrophages, under the influence of the local tissue cytokines, undergo phenotypic and functional differentiation (9, 10). This distinct phenotypes were historically subclassified into M1 and M2 (11).

Classical activation results in M1 macrophage phenotype, characterized by the production of high levels of pro-inflammatory cytokines, as well as an enhanced microbicidal capacity (12, 13). Alternative activation results in M2 macrophage phenotype, which have been reported responsible for wound healing and tissue remodeling (14, 15). Recently a more flexible classification has been suggested, in which macrophage types may overlap, and are considered a part of a continuum (16, 17).

Studies in human medicine have identified the pro-inflammatory cytokines IL-1β, IL-6, IL-8, and TNF-α, as main contributors in the course of the pathophysiologic inflammatory cascade of intervertebral disc diseases and the development of neuropathic pain (1). Similar studies in veterinary medicine show controversial results (2, 3), so it remains unclear which phenotype of macrophages may be dominant in the canine IVD herniation. A mixed-phenotype macrophage population has been identified within human degenerated IVD (4).

The purpose of this study was to characterize the phenotype of the Mϕ population present in naturally occurring IVD herniation. We hypothetize that in the acute phase of the disease corresponding to the time of prelevement of the disc material M1 (pro-inflammatory) macrophages will be the dominant cell population.

## Materials and Methods

### Study and Control Groups

The population of interest included client-owned chondrodystrophic dogs with acute thoracolumbar IVDH Hansen type I presented at our referral institution. Neurological signs were considered acute if they were observed for <48 h and they were graded according to a modified Frankel score: Paraplegia with no deep nociception (Grade 0), Paraplegia with no superficial nociception (Grade 1), Paraplegia with nociception (Grade 2), Non-ambulatory paraparesis (Grade 3), Ambulatory paraparesis (Grade 4), Spinal hyperestesia (Grade 5).

Data including signalment, onset of clinical signs, previous treatment, neurological examination, diagnosis and course of the disease was recorded.

The negative and positive control group samples were obtained from neurologically sound dogs that were euthanized for reasons unrelated to this study. Dogs included in these groups had no history of spinal disease and had not been under any kind of anti-inflammatory treatment.

Negative control samples consisted of nucleus pulposus material and positive control samples consisted of liver and mesenteric lymph nodes. Both the negative and positive control samples were obtained from the same group of dogs. All sample collections were approved by the local animal welfare authority (TVB Be 70/13).

### Diagnostic Imaging and Sample Processing

Confirmation of IVDH and extrusion site were determined with magnetic resonance imaging (MRI) (Philips Panorama HFO, 1.0-T open system, Philips Medical Systems Nederland B.V., The Netherlands) for all clinical patients. Following MRI, they all underwent standard decompressive surgery.

A standardized anesthetic protocol was conducted for all study group patients (18) and consisted of premedication with fentanyl before induction with diazepam (0.3 mg/kg IV) and propofol (to effect IV). Anesthesia was maintained with isofluorane in 100% oxygen adjusted according to the anesthetic depth. Analgesia was provided by fentanyl constant rate infusion (5 μg/kg/h). Preoperative antibiotics (cefazolin; 20 mg/kg IV) were administered 30 min before incision and repeated 120 min thereafter until the end of the surgical procedure.

A standard hemilaminectomy and fenestration through a dorsolateral approach was performed at the extrusion site. The extruded material was collected for immunohistochemistry.

All samples in the positive and negative control groups were surgically collected immediately after euthanasia.

All samples were embedded in OCT immediately after collection, shock frozen in liquid nitrogen and stored at −80°C until the day of processing.

Eight micrometer cryostat sections were prepared, air dried and stored at −80°C until the day of staining. All samples were immunostained without prefixation, permeabilization or blocking. The list of the primary antibodies is presented on Table 1. CD14 was used as marker Mϕ, MHCII for M1Mϕ and CD206 for M2Mϕ (19). On the day of staining the samples were first rehydrated with PBS. For CD14 and MHCII staining samples were first incubated with primary antibody (1:100 dilution, over night, 4°C), washed three times in PBS for a total of 2 min and afterwards incubated with the secondary antibody (Alexa 647 goat anti-mouse. Thermo Fisher Scientific (Waltham, Massachusetts, USA) conjugated with a fluorescence marker (Dilution 1:500, 45 min, RT). CD206 was already coupled with a conjugated antibody (PE) and therefore only a one step staining was required (1:20 dilution, over night, 4°C). Thereafter all samples were washed again with PBS three times for a total of 2 min and stained using Hoechst stain (1:1000 dilution, 15 min RT) followed by a last wash step in PBS as explained before. Finally, all samples were mounted for examination.

**Table 1:** List of primary Monoclonal antibodies used for surface labening.

**Clone**	**Antigen**	**Target Species**	**Source**
DG-CAM36A	CD14	Canine	Monoclonal Antibody Center^a^
3.29B1.10	CD206	Human	Beckman Coulter^b^
DG-TH81A5	MCHII	Canine	Monoclonal Antibody Center^a^

a *Monoclonal Antibody Center, Pullman, WA, USA*.

b *Beckman Coulter, Roissy, France*.

Patients samples were stained first with either CD14 combined with MCHII or CD14 combined with CD206, and subsequently with the combination CD206 plus MHCII.

All slides were examined using fluorescence microscopy and semi-quantitatively quantified using the following scale: (–) negative; (±) weak (<5% positive cells); (+) moderate (5–25% of positive cells); (++) strong (more than 25% positive cells) (3).

## Results

A global summary of the data and results is presented in Table 2.

**Table 2:** Global summary of the patient's data.

**Breed**	**Age (years)**	**Sex**	**Neurologic Grade at admission**	**Localization of Herniation**	**MRI most relevant findings**	**Surgical findings (subjective)**	**Neurologic Grade at discharge**	**CD14 positive cells**	**MHCII positive cells**	**CD206 positive cells**	**MCHII and CD206 positive cells**
Dachshound	4	M	3	Th13-L1	Disc extrusion, moderate compression Th13-L1 left. High intramedullary signal Th11-12	Mostly mineralized material	4	Moderate	Moderate	Negative	Negative
Dachshound	2	M	2	Th12-13	Th12-13 extrusion, right sided	Cottage cheese consistency material mixed with old blood	3	Weak	Negative	Weak	Negative
Dachshound	3	M	3	Th13-L1	T13-L1 extrusion, right sided	Fibrous and cottage cheese consistency	4	Moderate	Moderate	Moderate	Negative
Dachshound	5	F	3	L3-L4	L3-4 extrusion Abnormal signal of the liquor cranial to the extrusion site. TW2 hypointense	Massive amount of disc material. Mixed consistency	4	Moderate	Moderate	Moderate	Weak
Dachshound	5	M	4	L1-L2	L1-L2 extrusion. Multiple disc degeneration	Large amount of material Subdural bleeding Old bleeding	4	Moderate	Weak	Moderate	Weak
French Bulldog	5	F	3	L1-L2	L1-L2 extrusion	Fibrous and calcified material recovered	3	Moderate	Moderate	Moderate	Negative
French Bulldog	4	F	4	L2-L3	L2-3 extrusion with compression Generalized disc degeneration L1-2 and L2 through L4 spinal cord signal changes, increased T2W signal	Large bleeding and huge amount of compressive material	4	Moderate	Moderate	Moderate	Weak
French Bulldog	6	M	3	Th13-L1	Th13-L1 extrusion with large hemorrhage	Large hemorrhagic component	4	Weak	Weak	Weak	Negative
French Bulldog	2	F	3	L3-L4	L3-L4 extrusion, right sided	Mix of soft and calcified material. Small amount of active bleeding.	4	Moderate	Moderate	Moderate	Negative
Lhasa Apso	4	M	3	L1-L2	L1-L2 extrusion. Multiple disc degeneration	Mostly fibrous material recovered	3	Moderate	Moderate	Weak	Weak
Lhasa Apso	4	F	4	Th13-L1	Multiple disc degeneration Disc extrusion Th13-L1	Large amount of blood mixed with disc material	4	Moderate	Weak	Moderate	Weak
Shih Tsu	6	F	4	L1-L2	L1-L2 extrusion with severe spinal cord compression	Blood mixed with disc and fat	5	Moderate	Moderate	Weak	Negative
Shih Tsu	3	M	2	Th13-L1	Disc extrusion Th13—L1 and L1-2 mild protrusion	Mix consistency of herniated material	3	Moderate	Moderate	Weak	Weak
Pug	4	M	4	L1-L2	L1-L2 extrusion	Mostly calcified and hard consistency material recovered	4	Moderate	Moderate	Moderate	Negative
Pug	2	F	3	Th13-L1	Hernia originating from Th13-L1 expanding cranially to Th12 Generalized disc degeneration	Mix of soft and calcified material mixed with old blood	4	Weak	Weak	Negative	Negative

Fifteen dogs with surgically confirmed thoracolumbar IVDH were included in the study. Breeds included dachshounds (5), french bulldogs (4), lhasa apso (2), shih tzu (2), and pugs (2). There were 8 males and 7 females. Mean age 3, 9 years. Two patients were presented with neurological grade 2, eight with grade 3 and five with grade 4. Disc herniations were located in Th12−13 (1), Th13-L1 (6), L1-L2 (5), L2-L3 (1), and L3-L4 (2). A summary of the data is presented in Table 2.

The negative control group included 10 Beagles, and the positive control group included five.

**Figure 1:**
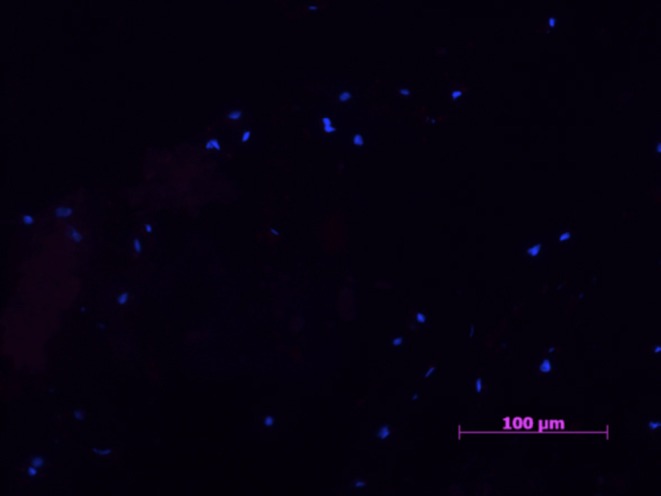
Example of a negative control samples with cell nucleus staining (HOESCH) and immunostained with CD206. No CD206 positive cells were found in any of the negative control samples 40X.

**Figure 2:**
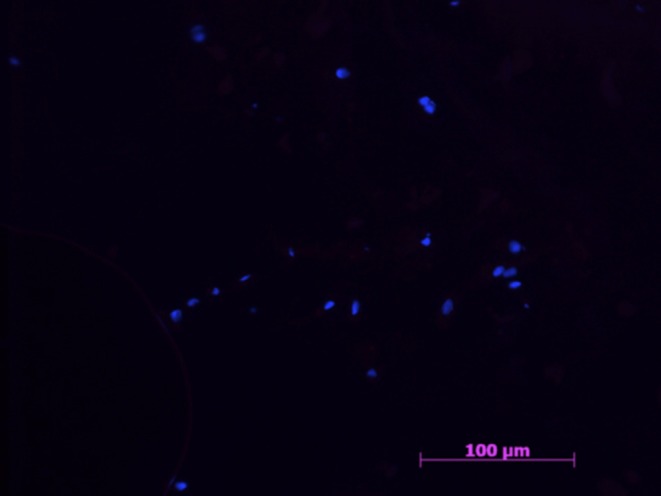
Example of a negative control sample with cell nucleus staining (HOESCH) and immunostained with MHCII. The picture shows the absence of MHCII positive cells, as well as a random distribution of the present cells 40x.

All samples in the negative control group were negative for all markers (Figures 1, 2).

A sparse amount of Hoechst stained nuclei were visible, always in an unorganized distribution.

The positive control group displayed several MHCII and CD206 positive cells throughout the histologically normal parenchyma, all of them being simultaneously positive to CD14. No cell was found with simultaneous CD206 and MHCII markers.

**Figure 3:**
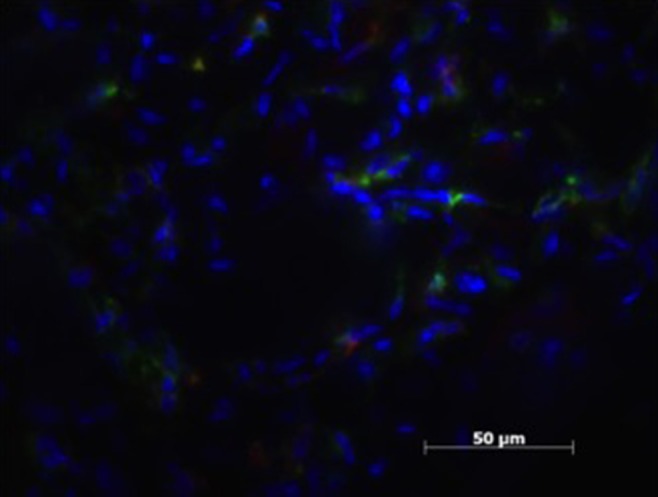
Patient sample with cell nucleus staining (HOESCH) and dual-immunostained for CD206 (red) and MHCII (green), displaying a majority of MHCII positive cells and only a few positive for CD206.

**Figure 4:**
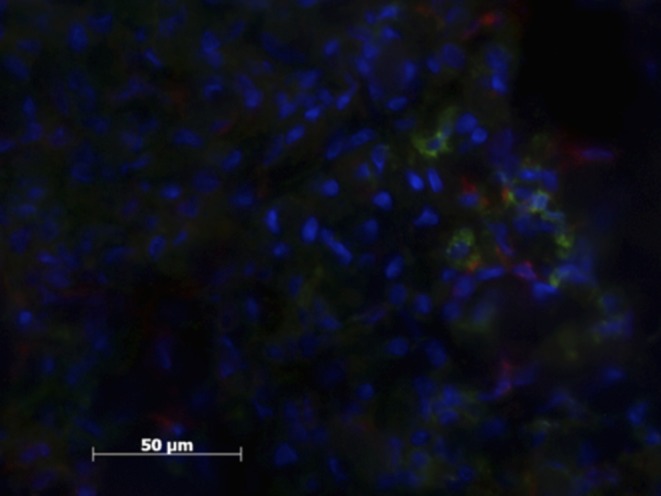
Patient sample with cell nucleus staining (HOESCH) and dual-immunostained for CD206 (red) and MHCII (green). This patient shows a larger proportion of CD206 positive cells in comparison with the patient shown in Figure 3.

All herniated samples displayed a mixed population of CD206, MHCII positive cells, that ranged in our scale from weak to moderate (see Table 1, Figures 3, 4).

The sample quality varied amongs individuals, with samples being very calcified, others containing only soft material and some containing parts of the annulus.

**Figure 5:**
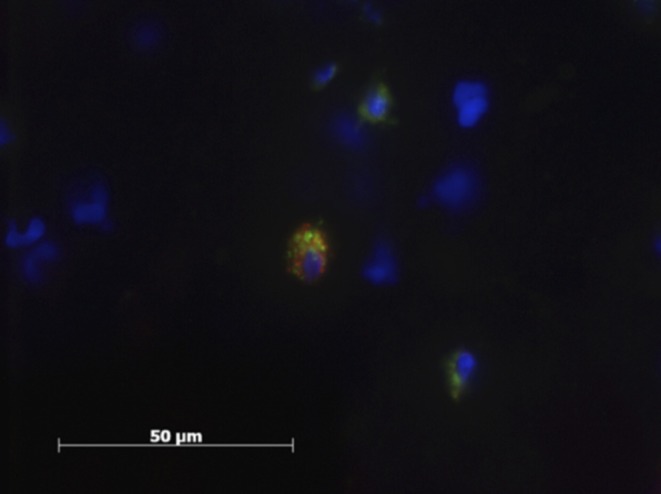
Patient sample with cell nucleus staining (HOESCH) and dual-immunostained for CD206 (red) and MCHII (green). The picture shows one of the double positive cells found in a patient sample.

Less than 5% of the Mϕ displayed markers for both M1 and M2Mϕ simultaneously (Figure 5).

## Discussion

This study describes the macrophage population resident in the herniated intervertebral disc, based on its phenotype. A weak to moderate mixed population of macrophages was uniformly present in all patients.

The intervertebral disc can be divided into four anatomic regions: annulus fibrosus, nucleus pulposus, cartilaginous endplates, and transition zone (20).

Under physiologic circumstances the nucleus pulposus has a high water content and very low cellular density, with water representing 80% and cells representing around 1–2% of its tissue volume in human beings (5, 6). The cell population consists of chrondrocytes, fibroblasts and notochordal cells. The latter group, the most abundant, distribute forming clusters (5, 21–25). All of our negative control group samples consisted of a few sparse nuclei, randomly distributed. The lack of cluster grouping is most probably attributable to sample processing, and not early degenerative changes considering that all negative control group samples were negative to CD14, MHCII, and CD206 markers. These findings are consistent with nucleus pulposus of healthy dogs.

Degeneration of the intervertebral disc disease is a multifactorial process that involves changes in cell composition as well as the extracellular matrix. The IVD undergoes cellular changes and large notochordal cells are lost and replaced by chondrocyte-like cells. Concurrently there is a change in the extracellular matrix, consisting of disorganized collagen fibers (21, 22, 25, 26). A difference in the lifespan of these large notochordal cells between chondrodystropic and non-chondrodystropic dog breeds has been reported in some studies. Large notochordal cells in non-chondrodystropic breed dogs may span into late adult life whereas they disappear quickly after birth in chondrodystrophic dogs (22). Cappello et al. reported a 13% of large notochordal cells in young chondrodystrophic breeds that dramatically reduced to 0.4% in adults, replaced by chondrocyte-like cells (26). This change in disc cell phenotype correlates with the grade of disc degeneration (20). However, a recent study (7) reported a lack or reduced number of these notochordal cells in sample of chondrodystrophic dogs with acute IVD extrusion. A clear predominance of inflammatory reaction was seen at the epidural site of disk extrusion in that study group. Interestingly, the mononuclear inflammatory infiltrate included a majority of macrophages and monocytes while T and B cells were sparse. A mononuclear population consisting mostly of macrophages has also been reported in human IVD extrusion (27–30). Furthermore, IVD-macrophage interaction is considered to have a major role in sciatica (31). Our herniated samples displayed an increased cellularity compared with the negative samples. They revealed a moderate amount of CD14 positive cells, which is in accordance with previous studies in human and veterinary medicine (7, 17). Markers for other inflammatory cells were not used and therefore it remains unknown if macrophages were the most abundant inflammatory cell in our study.

Macrophages are inflammatory cells that act both as drivers as well as regulators. They are profoundly influenced by their microenvironment; under the influence of cytokines they are able to differentiate into subgroups with different phenotype and function (32). Although there is considerable plasticity between distinct cell types, Mϕ have been historically classified as classically activated (M1) or alternatively activated (M2) Mϕ. Polarization into M1 Mϕ leads to strong antimicrobial activity and pro-inflammatory response. In contrast, M2Mϕ exibit anti-inflammatory function and regulate wound healing (11, 13, 17).

M1 cells were identified in our samples. A variety of inflammatory mediators such as Tumor Necrosis Factor (TNF)-α, Interleukin (IL)-6 and IL-1B have been previously implicated in the IVD degeneration in humans (1, 33). Similar studies in veterinary medicine have reached conflicting results. One study was unable to detect these same cytokines within the herniated IVD, but could demonstrate increased levels of Prostaglantin E2 and CCL2 in herniated disc compared with normal discs (34). In a second study IL-6 and TNF-α, were found to be up-regulated and IL-1β down-regulated in dogs with spontaneous IVD herniation (3).

However, others have reported a downregulation of pro-inflammatory genes in the epidural compartment after IVD extrusion. The authors concluded that, in fact, the epidural reaction was dominated by macrophages with tissue-remodeling functions (2).

Interestingly, M2 cells were found in a similar proportion to M1 in our patient samples. Fadda et al. reported an inflammatory infiltration of the extruded material with a pattern ranging from scattered neutrophils to well-organized granulation tissue. In that study chronic inflammatory patterns were observed in patients with an acute clinical history. Their authors hypothesized that the reported inflammation may develop even before the onset of clinical signs (7). The presence of CD206 positive cells in our samples is in agreement with their hypothesis. Although these patients presented with an acute onset of neurological signs, it is not uncommon for these patients to show previous episodes of back pain that often are unnoticed by the owner or are misread as abdominal pain.

M2 macrophages are key cells in tissue repair and remodeling. They are known to undergo phenotypic and functional changes, play complex roles in tissue repair as well as fibrosis and tissue regeneration (32, 35). There are signs that these M2 macrophages are implicated in IVD extrusion. In fact, spontaneous resorption of a herniated disc has been documented several times in the literature (36–42). The inflammatory response has been suggested to play a major role in this phenomenon. Although the exact resorption mechanism remains unclear, several studies have documented that macrophages and their high matrix metalloproteinases expression may play an important role (43, 44).

Some cells in the herniated sample group were positive to both MHCII and CD206. Recent research in human medicine examined the phenotype of the entire IVD macrophage population. In that study CCR7 was used as marker for M1 macrophage, CD206 for M2a and CD163 was used for M2c. The authors reported variation in the number of positive cells found per patient. Interestingly most of the positive cells were simultaneously positive to M1 and M2c markers in dual staining. The authors suggested that the studied macrophages might have been the same population of cells, and not distinct populations as they had hypothesized. They considered presence of CD206 positive cells as their next step in the investigation (4). In our samples, only few cells displayed simultaneously both markers, and therefore it is likely that there were in fact two macrophage populations. It is now known that the first M1/M2 classification approach does not adequately describe the spectrum of macrophage populations *in vivo*. Macrophage plasticity and switching in response to a changing environment has been evidenced in several studies that have questioned the *in vitro* oversimplified environments in which macrophages are stimulated with single cytokines (45, 46). Furthermore, Nakazawa et al. (4) reported differences in the macrophage distribution amongst the different parts of the IVD. These differences were correlated with the severity of disc degeneration, with more macrophages present in the nucleus pulposus of the most severely degenerated discs. Similarly, Fadda et al. reported that the severity of epidural inflammation correlated with the degree of nucleus pulposus calcification and epidural hemorrhage, which, in turn vas inversely correlated with the ability to regain ambulation (7). The samples in our population demonstrated a very homogenous macrophage population. Moreover, the number of patients was low, so similar conclusions could not be reached.

One of the main limitations of this study is the fact that we could not accurately count the number of positive cells of each category, providing M1:M2 ratios and instead a percentage estimation was given, as published previously (3). This is due to the fact that the positive cells were at times aggregating and therefore an accurate number of them could not be given. Considering the non structured consistency of the material recovered during surgery, the authors believe that immunohistochemistry might not be the best method for counting positive cells. Instead other techniques such as flow cytometry could be attempted in order to obtain more accurate numbers and thereafter be able to perform statistics with the obtained results (47).

Furthermore, other staining's such as H&E could have added more value to our research, for instance correlating our findings with those reported by Fadda et al. (7). For a more accurate description of the origin of these macrophages, however, a immunohistologic study of the whole intervertebral disc unit such as the one reported by Nakazawa et al. (4) would certainly provide more information. The recovered material during a decompressive surgery is often a mix of hemorrhage, different parts of the intervertebral disc as well as granulation tissue. Due to the sampling technique used during decompression surgery it was impossible to determine where our positive cells were located and their true distribution within the tissue.

Another limitation our study is the variability between the sampled material of our patients. The percentage of material recovered during decompressing surgery was variable between patients in quantity and quality. While some have material that is mineralized in consistency even mixed with pieces of annulus fibrosus and granulation tissue, others have herniated material mixed with blood and epidural fat, these differences are consistent with similar studies (7). The quantity varies as well, since not all the material that might have herniated can be collected during decompressive surgery. Also, the total amount of disc before herniation cannot be calculated using stereologic methods. Due to this limiting fact, the total number of macrophages cannot be calculated and only a semi-quantitative description could be performed, similarly to other studies (3). Nevertheless, despite the heterogenicity of the obtained samples, the results suggest a similar progression of the disease in all of our patients.

Finally, a larger number of markers would be needed to fully characterize our cell population. The double staining used in this study is not sufficient to fully ascertain that these are in fact M1 or M2 macrophages. Moreover, there is an ongoing controversy whether these cells might represent exogenous macrophages or resident intervertebral disc cells with an acquired phagocytic potential, in the process of differentiation from monocytes to macrophages (8, 48). In fact, other immune-privileged organs have been shown to contain an own immune-cell population. In the case of the intervertebral disc, although some nucleus pulposus cells seem to show phagocytic activity, it remains unclear if their action requires extraneous immune-cell assistance (49).

In conclusion, our study revealed a mixed phenotype population of macrophages that evidences the plasticity and dynamic complexity of Mϕ subpopulations as well as the chronic component of IVD disease despite its acute clinical presentation. Further research is required to determine if therapeutic strategies modulating the number of type of macrophages have a potential role in treatment of IVD herniation in dogs.

## Data Availability Statement

All datasets generated for this study are included in the article/supplementary material.

## Ethics Statement

The animal study was reviewed and approved by Animal Research Committee: Tierversuchsbewilligung TVB Be 70/13. Written informed consent was obtained from the owners for the participation of their animals in this study.

## Author Contributions

FF contributed to the conception and design, interpretation of data, critical revision of the manuscript for important intellectual content, obtaining funding, administrative, technical or material support, supervision, and final approval of the version to be published. AS contributed to the analysis and interpretation of data and critical revision of the manuscript for important intellectual content. MS contributed to the conception and design, analysis and interpretation of data, and critical revision of the manuscript. HM contributed to the conception and design, acquisition and data, analysis and interpretation of data, and critical revision of the manuscript. NV contributed to the conception and design, acquisition and data, analysis and interpretation of data, drafting of the manuscript, critical revision of the manuscript for important intellectual content, and obtaining funding. All authors agreed to be accountable for all aspects of the work in ensuring that questions related to the accuracy or integrity of any part of the work are appropriately investigated and resolved.

### Conflict of Interest

The authors declare that the research was conducted in the absence of any commercial or financial relationships that could be construed as a potential conflict of interest.
